# The ‘vicious cycle’ of personalised asthma action plan implementation in primary care: a qualitative study of patients and health professionals’ views

**DOI:** 10.1186/s12875-015-0352-4

**Published:** 2015-10-21

**Authors:** Nicola Ring, Hazel Booth, Caroline Wilson, Gaylor Hoskins, Hilary Pinnock, Aziz Sheikh, Ruth Jepson

**Affiliations:** School of Health Sciences, University of Stirling, Stirling, Scotland UK; Nursing, Midwifery and Allied Health Profession Research Unit, University of Stirling, Stirling, Scotland UK; Asthma UK Centre for Applied Research, Usher Institute of Population Health Sciences and Informatics, The University of Edinburgh, Edinburgh, Scotland UK; Scottish Collaboration for Public Health Research and Policy, West Richmond Street, Edinburgh, Scotland UK

**Keywords:** Barriers, Implementation, Personalised asthma action plans, Primary care, Qualitative, Self-management plans

## Abstract

**Background:**

Personal asthma action plans (PAAPs) have been guideline recommended for years, but consistently under-issued by health professionals and under-utilised by patients. Previous studies have investigated sub-optimal PAAP implementation but more insight is needed into barriers to their use from the perspective of professionals, patients and primary care teams.

**Methods:**

A maximum variation sample of professional and patient participants were recruited from five demographically diverse general practices and another group of primary care professionals in one Scottish region. Interviews were digitally recorded and data thematically analysed using NVivo.

**Results:**

Twenty-nine semi-structured interviews were conducted (11 adults with asthma, seven general practitioners, ten practice nurses, one hospital respiratory nurse). Three over-arching themes emerged: 1) patients generally do not value PAAPs, 2) professionals do not fully value PAAPs and, 3) multiple barriers reduce the value of PAAPs in primary care. Six patients had a PAAP but these were outdated, not reflecting their needs and not used. Patients reported not wanting or needing PAAPs, yet identified circumstances when these could be useful. Fifteen professionals had selectively issued PAAPs with eight having reviewed one. Many professionals did not value PAAPs as they did not see patients using these and lacked awareness of times when patients could have benefited from one. Multi-level compounding barriers emerged. Individual barriers included poor patient awareness and professionals not reinforcing PAAP use. Organisational barriers included professionals having difficulty accessing PAAP templates and fragmented processes including patients not being asked to bring PAAPs to their asthma appointments.

**Conclusions:**

Primary care PAAP implementation is in a vicious cycle. Professionals infrequently review/update PAAPs with patients; patients with out-dated PAAPs do not value or use these; professionals observing patients’ lack of interest in PAAPs do not discuss these. Patients observing this do not refer to their plans and perceive them to be of little value in asthma self-management. Twenty-five years after PAAPs were first recommended, primary care practices are still not ready to support their implementation. Breaking this vicious cycle to create a healthcare context more conducive to PAAP implementation requires a whole systems approach with multi-faceted interventions addressing patient, professional and organisational barriers.

**Electronic supplementary material:**

The online version of this article (doi:10.1186/s12875-015-0352-4) contains supplementary material, which is available to authorized users.

## Background

Personalised asthma action plans (PAAPs) also known as self-management plans were first recommended for patients over 25 years ago [1, 2]. These paper or electronic plans are intended to assist patients/parents in their asthma management by reinforcing guidance on what to do when asthma worsens. There is overwhelming evidence these plans can, if appropriately used, improve treatment adherence and patient outcomes – for example, reducing asthma exacerbations and hospitalisations [3–5]. PAAPs continue to be recommended by national and international guidelines as core components of supported self-management [6, 7]. However, there is considerable, longstanding international evidence indicating that PAAP implementation (issuing and review by professionals and use by patients/carers) is sub-optimal [8–11] and needs to be improved if their clinical benefits are to be maximised.

We previously systematically reviewed the quantitative [12] and qualitative literature [13] investigating PAAP implementation in primary care and synthesised the findings [14]. This work identified interventions that were effective in increasing PAAP ownership by patients/parents, but provided little information on how to embed their use into routine self-management practice longer-term [12]. It also highlighted that these plans need to be more patient-centred with greater partnership working and more effective communication between patients/parents and professionals during PAAP development [13]. This evidence synthesis identified that in order to develop a future intervention to promote PAAP use in primary care there was need to obtain further insight into PAAP implementation in primary care from the perspective of patients and professionals. This paper reports the findings of a qualitative study exploring patients’ and primary care professionals’ experiences of PAAP use, their views on the role of these plans in asthma management and suggestions on how their use could be better integrated into routine care in future.

## Methods

Prior to the project commencing ethical approval was obtained from the appropriate East of Scotland Research Ethics Committee (REC1) and the study was approved by the relevant National Health Service (NHS) Research & Development Committee. Our methods are outlined below according to the consolidated criteria for reporting qualitative research (COREQ) three domains [15].

### Research team

The multi-disciplinary research team included primary care professionals and experienced qualitative researchers. A public health nurse (CW) carried out the interviews after full training. The research team was supported by a project Steering Group which included primary care practitioners with an interest in asthma care.

### Study design

The study involved content analysis of semi-structured one-to-one interviews with adults with asthma living at home and primary care health professionals. One Scottish NHS Board area (*n* = 14) was the setting for this study. This medium sized mainland region included urban and rural areas of varying socio-economic levels. Every general practice (*n* = 57) in the area was sent a study invitation letter and information pack. Seven practices were interested in taking part in the study and five were recruited. Practices were selected using specific criteria to ensure they represented a range of differing clinical contexts including practice size, training and non-training practices, urban and rural settings. Participating practices were selected from across the NHS Board area to ensure inclusion of patients living in varying circumstances such as those in socially isolated or deprived areas. Selected practices provided written consent and practices were reimbursed for costs associated with searching asthma registers. Adults with asthma and professionals were recruited as follows.

- *Adults with asthma:* Administrative staff from participating practices identified a purposive sample of 20 patients from each practice asthma register. Eligible patients had to have a diagnosis of asthma; have been issued with an asthma prescription in the past year; have the capacity to consent; and speak enough English to be interviewed. To maximise variation, practices identified male and female patients with asthma of any severity from four age groups i.e. 16–25, 26–45, 46–65 and >66 years. One hundred patients were sent study information and invited to take part. Twenty patients were interested in being interviewed and returned completed expression of interest forms with brief demographic details such as their age. The research team selected up to three patients per practice offering a range of gender, age group, length of asthma diagnosis and whether or not they had a PAAP. (A maximum of three patients per practice was a pragmatic decision based on balancing the capture of in-depth patient views within the project timescales and budget). CW contacted selected patients to provide further information about the study and gain their written consent. Patient participants received a £10 shopping voucher post-interview.
- *Health professionals:* Patient interviewees were asked to name the primary care (and any other health) professionals with whom they consulted most often about their asthma. All named professionals were sent project information and invited for interview. Two professionals from the participating practices were unavailable for interview so an equivalent professional from the same practice was invited to participate. To ensure we obtained a breadth of professional views we recruited an Additional group of Practice Nurses (PNs) and General Practitioners (GPs). Individuals (*n* = 6) in this sixth group worked in the same NHS area, but were not affiliated to the five participating practices. These individuals were purposively selected as they were known within the NHS locally for their interest in asthma. CW contacted all identified professionals to obtain written consent pre-interview. PNs and GPs received reimbursement towards locum costs.

Interviews were conducted using a topic guide (see Table 1) devised with our clinical partners. This was piloted prior to use with patient and professional representatives known to the researchers and refined accordingly. Interviews lasted 30–60 min, were digitally-recorded and field notes were taken. Patients were interviewed at home, and health professionals in their workplace at a convenient date and time. Interviewees were shown a selection of PAAP templates at interview, including those produced by Asthma UK [16] and those promoted by the local NHS Board, and were asked to confirm which plans, if any, they owned or issued. Interviews were transcribed verbatim. Interview transcripts were checked for accuracy (NR/CW), their content anonymised and then imported into NVivo 10. To preserve anonymity and confidentiality, participants were assigned an individual project code known only to NR, CW and HB. All data were stored securely and password protected.

**Table 1:** Summary of patient and professional interview topic guides

Patient interview topic guide:
Background information e.g. length of time with asthma? Attended for asthma review in last year? Asthma related hospital admission in last year?
Have they ever been issued with a Personal Asthma Action Plan (PAAP)? What type was it? How was it issued and by whom? Do they use it? How?
Do they think PAAPs have a role in managing asthma? How? When? Do they need or want one?
What are the barriers to the use of PAAPs by patients? What would encourage the use of such plans in the future?
Health professional interview topic guide:
Background information e.g. their role and asthma education/qualifications.
Have they ever issued a PAAP? How, when and to whom? Do they ever review these? What type(s) of plans do they issue/review? What type(s) of PAAPs are they familiar with?
Do PAAPs have a role in asthma management? If so, how? When? For whom? What are the barriers to professionals issuing and/or reviewing these in primary care? How could PAAP use be encouraged in future?

### Analysis

Patient and professional interview data were content analysed to identify emergent themes. NR, CW and GH read and re-read initial interview transcripts to identify preliminary themes such as participants’ experiences of PAAPs and barriers to their use. These initial themes formed a preliminary coding frame used to code data in the remaining transcripts. The coding frame was refined in discussion with the wider research team as analysis progressed. Interview transcripts were coded in NVivo by NR, CW and HB working in pairs to maximise reliability. Any coding disagreements were referred to the third coder for arbitration. Data saturation was reached when no new themes or sub-themes were identified by the coders within the interview transcripts – this occurred after eight patient and 12 professional interviews. Data on the type of PAAPs issued/reviewed by professionals and owned/used by patients within each of the five practices were extracted from the interview transcripts, transferred into matrices (Microsoft Excel) and descriptively analysed to identify, for example, which PAAP formats were in circulation in the same practice.

## Results

In total, there were 29 participants (11 patients and 18 health professionals) (see Table 2). Eleven patients were interviewed – four men and seven women from five general practices (Table 2). The number of patients interviewed per practice ranged from one to three (Table 2). All but one patient was aged 40 years or over. Eighteen health professionals were interviewed (10 PNs, seven GPs and one hospital respiratory nurse). The 17 primary care professionals worked in nine different practices - approximately 15 % of the practices in this NHS area. The majority of health professional participants were women (*n* = 14) apart from four male GPs. The following themes emerged from the interview data and are reported below with other illustrative quotes in Additional file 1: Table S1.

**Table 2:** Study group, practice and participant information

	Gender	Age	Asthma duration (years)	Smoker: X = no ✓ = yes	Had asthma diploma	Career duration (years)	Participant numbers
Group 1: training practice, urban location, practice population < 3000							
Pat* 1	Male	46–65	<5	X	N/A	N/A	5
Pat 2	Female	26–45	5+	X	N/A	N/A	
Pat 3	Female	46–65	5+	Ex-smoker	N/A	N/A	
PN1	Female	N/A	N/A	N/A	✓	<10	
GP 1	Male	N/A	N/A	N/A	X	20+	
Group 2: urban & semi-rural location, practice population > 4000							
Pat 1	Female	46–65	5+	Ex-smoker	N/A	N/A	6
Pat 2	Male	46–65	5+	X	N/A	N/A	
PN 1	Female	N/A	N/A	N/A	✓	10–20	
PN 2	Female	N/A	N/A	N/A	✓	<10	
GP 1	Male	N/A	N/A	N/A	X	20+	
Hospital nurse 1	Female	N/A	N/A	N/A	Specialist		
Group 3: rural location, practice population < 3000							
Pat 1	Male	46–65	5+	X	N/A	N/A	5
Pat 2	Female	46–65	5+	✓	N/A	N/A	
Pat 3	Female	46–65	5+	X	N/A	N/A	
PN 1	Female	N/A	N/A	N/A	✓	20+	
GP 1	Female	N/A	N/A	N/A	X	20+	
Group 4: urban practice, practice population > 4000							
Pat 1	Female	46–65	5+	X	N/A	N/A	3
PN 1	Female	N/A	N/A	N/A	✓	20+	
GP 1	Female	N/A	N/A	N/A	X	10–20	
Group 5: training practice, urban & semi-rural, practice population >4000							
Pat 1	Male	46–65	5+	X	N/A	N/A	5
Pat 2	Female	66+	5+	X	N/A	N/A	
PN 1	Female	N/A	N/A	N/A	✓	<10	
PN 2	Female	N/A	N/A	N/A	✓	10–20	
GP 1	Male	N/A	N/A	N/A	X	10–20	
Additional health professionals group							
PN 1	Female	N/A	N/A	N/A	✓	10–20	5
PN 2	Female	N/A	N/A	N/A	✓	20+	
PN 3	Female	N/A	N/A	N/A	✓	10–20	
GP 1	Female	N/A	N/A	N/A	X	10–20	
GP 2	Male	N/A	N/A	N/A	not known	10–20	
Total participants: 11 patients + 18 professionals							29

Notes: *For abbreviations - see list of abbreviated terms in main text

Patients had asthma of any severity. No patient had had an asthma related hospital admission in the last year

Number of patients interested in taking part/Number interviewed per practice: Group 1: 4/3; Group 2: 4/2: Group 3: 7/3: Group 4: 1/1; Group 5: 4/2. Patients were chosen to provide the broadest range of views e.g. age, gender, with/without PAAPs

- Patients generally do not see the value of PAAPs
- Professionals do not fully value PAAPs
- Multiple multi-level barriers are reducing the value of PAAPs.

### Patients generally do not see the value of PAAPs

Six patients said they had been given a PAAP (Table 3), but only three patients had their plans with them at interview. Table 4 details the different PAAP formats/templates used by the participants. Regardless of the format of their PAAP, none of these six patients were using their plans, as illustrated below:

**Table 3:** Participants issuing, reviewing or owning Personalised Asthma Action Plans

Study group	Professional participants	Ever issued a PAAP*	Ever reviewed a PAAP	Patient participants	Indicated pre-interview had a PAAP	Indicated at interview had PAAP	PAAP shown to interviewer	PAAP ever been reviewed
Group 1	PN1	✓	✓	Pat 1	X	X	X	X
	GP1	X	X	Pat 2	X	X	X	X
				Pat 3	✓	✓	X	X
Group 2	PN1	✓	X	Pat 1	✓	✓	✓	X
	PN2	✓	✓	Pat 2	X	✓	✓	X
	GP1	✓	X					
	HN1	✓	X					
Group 3	PN1	✓	X	Pat 1	X	X	X	X
	GP1	✓	X	Pat 2	✓	✓	✓	✓
				Pat 3	✓	X	X	X
Group 4	GP1	X	X	Pat 1	X	✓	X	X
	PN1	✓	✓					
Group 5	PN1	✓	✓	Pat 1	X	✓	X	X
	PN2	✓	✓	Pat 2	X	X	X	X
	GP1	X	✓					
Other	PN1	✓	X	N/A	N/A	N/A	N/A	N/A
	PN2	✓	✓					
	PN3	✓	X					
	GP1	✓	X					
	GP2	✓	✓					
Total	*n* = 18	15 (83 %)	8 (44 %)	*n* = 11	4 (36 %)	6 (54 %)	3 (27 %)	1 (10 %)

*For abbreviations - see list of abbreviated terms in main text

**Table 4:** Types of Personalised Asthma Action Plans issued, reviewed and owned by study group

Study group	Type of Personalised Asthma Action Plans	
	*Owned by patient(s)*	*Issued or reviewed by professionals*
1	Peak flow based ‘wee handbook’ - type unknown. Patient could not recall and did not recognise it from the interviewer selection.	NHS symptom management plans
		Asthma UK plans
2	Local NHS long term management plan	Hand written on ‘bits of paper’
		NHS short and long term management plans
	Asthma UK plan	Asthma UK plans
3	Postcard sized peak flow plan – source unknown as no publisher details shown on plan	Asthma UK plans
		NHS symptom and long term management plans
		Handwritten plans
4	Desmond Dragon children’s plan	Hand written on note paper
		NHS short term management plans
		Asthma UK plans
5	Asthma UK plan	Asthma UK plans
Other	Not applicable	Handwritten on ‘bits of paper’ or on patient prescriptions
		Asthma UK plans
		Drug company asthma plans
		NHS local asthma plans (long term management)
	Across study groups 1–6	
Written plans owned by patients:		Written plans issued by professionals*:
Type unknown = 1		Handwritten (6 participants from 4 study groups) on paper (*n* = 4) or prescriptions (*n* = 2)
Peak flow based postcard type = 1		Asthma UK plans - 9 participants across all groups
NHS long-term management = 1		NHS asthma plans short, long-term or symptom management - 6 participants from 5 groups
Asthma UK plan = 2		Drug company plan - 1 participant from 1 group
Desmond Dragon = 1		*some professionals gave multiple answers

*‘I haven’t referred to my plan for a long time…. I keep it through there in a box’ (Patient 2/group 2)* (see Table 2 for participant details)*.*

These six patients recalled having little input into developing their plan and said their health professionals had told them what was going to be put in it and asked them to approve the content. Only one of the six patients issued with a PAAP reported ever having had it reviewed by a health professional (Table 3). Consequently, for these six patients their PAAPs were perceived as having little personal value because these were outdated and did not reflect their asthma symptoms, peak flow measurements and/or current treatment. For example, one patient reported having a specific physical skin sensation which indicated an asthma attack was imminent, but this was not included in her plan as an indicator for action (Patient 2/group 3). Another patient reported:

*‘The asthma plan said if your peak flow drops by a certain percentage call for a doctor. But, that is not right for me – my peak flow is so good on paper but at night I still go into spasm’ (Patient 1/group 4)*.

Overall, the 11 patient participants did not think that PAAPs were suitable or appropriate for use by all those with asthma. Typically, patients reported not wanting or needing a PAAP because:

*‘a) I don’t think my asthma is bad enough and b) I would know what to do’ (Patient 1/group 3).*

Whether a PAAP would be of value to others, depended on how long since their asthma was diagnosed, how severe it was, whether the person with asthma was literate, would *‘care enough to manage’* their condition and be ‘*motivated to use’* one *(Patient 1/group 3).* There was, however, support for their use by *‘vulnerable people*’ *(Patient 1/group 5)* including the newly diagnosed, children and pregnant women because these people were thought to be learning about their asthma and how to manage it. A PAAP was considered useful until these patients were able to:

*‘Know when their breathing goes a bit haywire to double up, or whatever, on their medication’ (Patient 2/group 3).*

### Professionals do not fully value PAAPs

Fifteen health professionals had issued a PAAP at least once (Table 3), but there was not wholehearted support for the use of these guideline recommended plans in primary care. Although one PN reported that PAAPs were *‘absolutely essential’ (PN 1/group 5)* in asthma management the following quotes were more typical of health professional participant views (see also Additional file 1: Table S1):

*‘I think action plans just don’t work for some people. You can give them a plan; they are not going to [use] it. So, really what’s the point in spending all that time giving them a plan when they don’t really need it? (GP1/group 4).*

*‘They are handy, they are obviously necessary but I wouldn’t say they are the be all and end all of asthma management … Although they are important to a degree, they don’t figure, to the highest thing that I do for a patient’ (PN1/group 2).*

*‘It goes straight into the bin’ (PN1/other professionals).*

Only two PNS reported routinely issuing PAAP to all those with asthma – other PNs issued these much less often, for example:

*Interviewer: ‘how many written plans do you issue?’ ‘Not nearly enough. I would say less than half. Probably 25 % perhaps but that’s just a guess’ (PN1/group 3).*

The health professionals interviewed did not routinely issue PAAPs because they did not consider these to be of value to all those with asthma. Overall, professional participants thought these plans were best suited to certain types of patients and, as such, issued them selectively to priority groups including children, those with more severe asthma, recent asthma exacerbations and/or those requiring changes to their medication. Reflecting patient views, professional interviewees also considered the newly diagnosed to be a priority group for receiving PAAPs however, these patients were not always issued with a plan immediately on diagnosis, as exemplified below:

*‘I wouldn’t give it [a PAAP] at the first appointment and maybe not even at the second appointment because … I feel that they’ve [patients] got too much information … So, I just let it all settle and maybe on their third appointment I give them their plan’ (PN2/group 2).*

Whilst 15 health professionals reported issuing a PAAP at some point, only eight (six PNs and two GPs) reported having ever reviewed one (Table 3). Even when professionals reported reviewing PAAPs this was not done consistently for every patient with a plan. A key reason for professionals not reviewing PAAPs was patients not bringing these to their asthma consultations - only five professionals could recall patients ever doing this. The health professionals said patients kept their plans ‘*hidden away somewhere’* (*GP1/*g*roup* 3) and could not find them when they were needed. As one PN noted:

*‘[Patients] never have it with them … very, very rarely - maybe one now and again will come in with [it]…I can’t recall the last time I got somebody coming back with an asthma plan’ (PN1/group 2).*

Consequently, when the health professionals did review previously issued PAAPs this usually involved determining whether patients had retained their plans at home rather than a meaningful discussion of its content as illustrated below:

*‘I always make sure that they have still got it at hand … and they still know where it is’ (PN2/other professionals).*

### Multiple multi-level barriers are reducing the value of PAAPs

Patients and health professionals in this study all reported barriers to the issuing, review and use of PAAPs. Barriers were categorised as individual or organisational but these compounded to severely hinder PAAP implementation in primary care, reducing the value of PAAPs for professionals and patients. **Individual barriers** were patient and professional specific. Data providing insight into patient barriers fell into two main sub-themes - lack of awareness and PAAPs not meeting patients’ needs. First, lack of PAAP awareness - one patient, for example, diagnosed with asthma in the last five years (Patient 1/group 3) had never heard of these plans. Three patients stated pre-interview they did not have a PAAP and only recalled having been given one when they were shown a selection of these at interview (Table 3). Another patient interviewee reported:

*‘I’ve got a feeling I was given [one] but I can’t remember what it was or what it looked like’ (Patient 2/group 1).*

Second, PAAPS were not meeting the needs of patients. None of our patient participants wanted PAAPs that focused solely on medicines and *worsening* asthma because they felt able to manage these aspects of their condition. There was, however, some unmet need for asthma plans to help patients manage their condition at other times. For instance, four patient participants wanted plans giving them guidance on when to seek professional help for asthma symptoms which had *improved but persisted* such as, guidance on:

*‘When to go to your GP? Should I go just now? I’ve woken up every night for the last week [with a cough]. Should I go? Or would they [the GP] just say ‘Oh, what are they here for’?’ (Patient 2/group 1).*

Some patients wanted PAAPs that provided guidance on increasing their exercise levels (*n* = 2) or going abroad (*n* = 4) but these information needs were not recognised by the professional participants. Other examples of PAAPs not meeting patient needs were if they only included peak flow measurements and did not include symptoms, were not succinct, easy-to-read or looked like *‘a government health warning…– grey and old’ (Patient 1/group 5).* Patients also needed PAAPs tailored to their circumstances. As one patient commented a PAAP:

*‘Needs to feel as if it is something that you have written or put into place yourself and … that it relates to [you] specifically. [It is] not someone else saying, ‘oh right, this is what your asthma is like – this is your action plan’. It also helps [that] whoever is providing the advice … gives a much more personal focus on how your asthma manifests itself’ (Patient 2/group 1).*

Professional barriers also formed two main sub-themes. Professional attitudes and behaviours emerged as the first and largest sub-theme supported by data from all 29 participants. Two key examples of this barrier were professionals not reinforcing PAAP use and/or not involving patients in PAAP development. Twelve professionals did not routinely ask patients to bring their PAAPs with them to appointments or could not recall ever doing so. Professional participants observed that patients did not come to appointments ‘*brandishing*’ their plans’ *(GP1/group 1)* and that the clinical context did not facilitate review and discussion of PAAPs. For example:

*‘The only time I see a patient [with asthma] is when they are ill and they don’t mention [their plan] to me’ (GP1/group 2).*

*‘I never mention [PAAPs] [to patients] ever and it’s probably because I don’t know the patients who have a plan and those who don’t. So they [patients] never mention it to me either’ (GP1/group 4).*

Where the health professionals were issuing and reviewing PAAPs they did not all actively encourage patient involvement in their development. Although one PN (PN1/group 1) stressed the importance of patients and professionals making ‘*dual goals together’* and noted that sometimes patients disagreed with the information she wanted to include in their plan, her response was unusual. The following quotes illustrate that, more typically, patient participation in PAAP development was passive:

*‘Very often as we are talking, I am writing it and then I will go back over it and flip through the pages with them and tell them what I’ve written and why’ (PN1/group 4).*

*‘It’s a bit of paper … to back up what we are saying to [patients]’ (PN1/other professionals).*

PAAP specific barriers formed the second main sub-theme. Problems with PAAP content, format and/or accessibility caused practical difficulties for staff and hindered implementation. Five professional participants, for instance, reported that PAAP templates were not meeting their needs because they could not be easily personalised to individual patients, see below and Additional file 1: Table S1.

*‘There is no real room to give guidance about steps [as per the asthma guidelines]. It would be nice to say what action patients should take … when they have stepped-up [for example] how long do they need to be symptom free before they step back down?’ (PN1/group 1).*

*‘The local NHS asthma plan says ‘you’ve got to increase your preventer to two puffs but sometimes the patient isn’t taking a preventer – only a reliever’ (PN2/ group 5).*

### Organisational barriers

Data supporting this theme were provided by all professional participants. Practical barriers were frequently cited. Eight professionals reported difficulties easily obtaining paper PAAP templates in colour format. Three professional participants reported not knowing whether previously issued PAAPs were stored in patients’ records or had difficulty accessing these during their consultations. Another PN reported that individualised changes made to a patient's electronic PAAP were ‘lost’ or over-ridden whenever her computer system was updated. Six professionals said that consultation times were not long enough to discuss patients’ asthma symptoms, medications, inhaler technique and PAAPs. Consequently, as one PN noted:

*‘I am sometimes a bit half-hearted [about issuing PAAP] because I am remembering about the use of time [required]’ (PN1/other professional group).*

The second organisational barrier to emerge was that general practice processes for the issuing and review of PAAPs were fragmented. One key example, cited by three interviewees, was that practice letters inviting patients to attend for routine asthma review did not ask them to bring their PAAPs to these appointments. Consequently, these plans could not be reviewed unless they were accessible in the patients’ records, which was not always the case. Another example of fragmentation related to the many PAAP formats in circulation. Our professional participants (*n* = 15) reported using at least seven different formats of PAAP (Table 4). This variety caused problems within and between practices. Between general practices, PAAP were not *‘transferable’ (PN1/group 3)* when patients changed doctors and moved to other practices. Within practices it caused confusion as professional participants were not always certain which type of PAAPs their colleagues issued, if any, such as:

*‘I don’t know which plan my [PN] colleague uses… I don’t know if [the GPs] issue them I am not certain they do’ (PN2/group 5).*

The differing role of GPs and PNs in asthma management also contributed to fragmented PAAP processes. All professional participants considered PAAP issuing and self-management education to be primarily a nursing role within the context of asthma reviews. But, PAAPs were designed for use by patients when their asthma gets worse and at such times they see their GPs yet, only two GP participants reported reviewing these plans when patients presented to them with acute asthma at unplanned appointments (Table 3). Consequently, opportunities to issue and review PAAPs were lost, as one nurse commented:

*‘If [patients] have been to the doctor, the doctor then asks them to come back to me and at that point I should issue them. But, I have to say they don’t always come back to me’ (PN1/group 3).*

## Discussion

### Principal findings

**Fig. 1:**
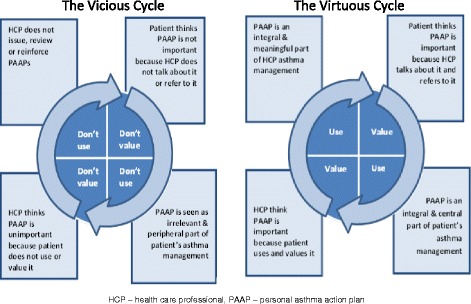
Cycles of Personalised Asthma Action Plan implementation in primary care

Over two decades after PAAPs were first advocated [1, 2], we have found that in primary care these are still not being routinely issued/reviewed by health professionals or used by patients. For our patient and professional participants, despite being recommended by guidelines [6, 7], PAAPs were not an integral part of everyday primary care asthma (self-) management. Instead, as one participant described these plans existed *‘in the ether’ (GP1/group 2)* of practice. Multiple individual and organisational barriers compounded to hinder PAAP implementation for our participants. These barriers included PAAP templates that are not fit for purpose or easily accessible; fragmented primary care processes for PAAP issuing and reviewing; and patients, PNs and GPs not being totally convinced about the usefulness and relevance of such plans. Our findings suggest that primary care PAAP implementation is caught within a *Vicious Cycle* (Fig. 1). Professional participants are not routinely issuing, discussing or reviewing PAAPs with all patients. Patient participants view PAAPs as having a peripheral or non-existent role in their asthma management. When previously issued PAAPs become out-of-date and redundant, patient participants do not talk about them or bring them to their appointments, reinforcing their professionals’ views that PAAPs have little value, meaning they are less likely to review existing plans or issue more, thereby perpetuating a cycle of sub-optimal implementation.

### Study strengths and limitations

Our study reports findings from the perspective of individual patients and professionals but by recruiting participants predominantly from five general practices our study was novel as we were able to explore PAAP implementation within primary care teams. Participating primary care teams represented various clinical settings including training/non-training practices and urban/rural areas. Patients were selected with a range of views and experiences including different ages/gender and length of asthma diagnosis. Due to ambiguity regarding PAAPs [17] participants were shown at interview a sample of plans they were likely to have seen, such as the Asthma UK plan [16], to ensure they all understood what we were referring to. This resulted in more patients saying at interview they had PAAPs than had indicated having one pre-interview. Including pictures of PAAP templates in the study invitations may have brought more patients forward for interview giving us a greater range of patients from which to select. Our findings are based on the research team’s interpretation of the interview data and reflect their backgrounds in primary care nursing, general practice and social science. The research team was supported by a multi-disciplinary project steering group including representatives from the local Airways Managed Clinical Network. Participants’ views may not be representative of other patient and professional experiences, for example our study excluded children, none of our patients were newly diagnosed and patients were recruited from only one geographical area. The patient participants formed a relatively small sample and they were not purposively sampled to include a range of factors that may influence PAAP use (such as educational levels, economic circumstances, co-morbidity or asthma severity).

### Interpretation of our findings and implications for practice

Sub-optimal PAAP implementation is just one example of the internationally recognised challenge of getting research into mainstream practice [18]. Generally, three overlapping elements – evidence, context and facilitation – are essential for successful implementation [19–22]. Our study provides *evidence* that for PAAP use to improve in primary care and their clinical benefits realised, simply increasing the number of patients issued with, and owning, such plans will not be enough to guarantee their use. There is a need to create a *Virtuous Cycle* of PAAP implementation, a primary care culture in which these plans are valued more by patients and professionals as purposeful and dynamic asthma management tools (Fig. 1). Changing professional attitudes and behaviours to achieve a Virtuous Cycle will be challenging because GPs and PNs were first reported as ‘unenthusiastic’ and ‘ambivalent’ towards PAAPs over 15 years ago [23] and our study reveals that indifferent professional attitudes towards PAAPs continue to exist in primary care. Yet, our participants were not totally indifferent to PAAPs, our patient and professional participants considered these plans to be of relevance to certain groups including the recently diagnosed, children and those with severe or unstable asthma. It is also important to note that many of our individual professional participants were actively promoting PAAPs but, their colleagues and general practices processes were not fully engaged in supporting their actions. Overall, however, our findings do indicate a general lack of ‘organisational readiness’ [24] to support PAAP implementation with health professionals (individuals and teams) not wholly committed to initiating and sustaining the change[s] required to promote PAAP use in primary care. This is a critical barrier to development of a Virtuous Cycle because a supportive *context* is so vital to successful implementation generally [22]. Addressing this fundamental, but under-acknowledged organisational barrier needs a whole systems approach [25] with ‘multi-faceted’ interventions directed towards *facilitating* change amongst patients, professionals and general practices [18, 26, 27]. This could be achieved in two main ways.

First, general practices need to systematically review their organisational processes so they can create clinical environments better able to support asthma plan implementation. Practices could, for example facilitate team-working and communication by explicitly agreeing on which asthma plan templates they will use and consider how these plans can best be reviewed when patients present with acute episodes. Practices could reinforce the need to issue/review PAAPs through electronic prompts in patients’ records and/or by administrative staff asking patients booking unplanned asthma appointments to bring their plans with them. As lack of time is a barrier to PAAP use, it is essential professionals can access these plans quickly during consultations and that asthma plan templates are more flexible in format so their content can be easily personalised for individual patients. Indeed, it is likely that a range of suitable templates will be needed to suit different asthma phenotypes and endotypes as well as the range of triggers and diversity of patient preferences. Patients could also be encouraged to bring partially completed plans with them to their annual asthma reviews – an approach previously found beneficial [28]. Whilst currently there is insufficient evidence of the effectiveness of patient-held smartphone and tablet apps in asthma self-management [29] such mobile technology can facilitate electronic access to asthma plans by patients/parents and professionals during consultations [30]. General practices could also set ‘realistic implementation goals’ [31], for example, targeting PAAP issuing/review towards those groups, such as the newly diagnosed, that are perceived by patients and professionals as a priority for receiving them.

Second, increasing the perceived relevance of PAAPs to patients/parents and professionals could also promote development of a Virtuous Cycle. PAAPs lacking in relevance for professionals and patients was first recognised over a decade ago [23] so a fresh approach is required to overcome this barrier. For patients, improving PAAP design and accessibility [32–34] should bring some benefits, but our patient participants with asthma of longstanding duration indicated a need for PAAPs better suited to their requirements. In future, *Living with Asthma* plans addressing a broader range of self-management issues [17], such as what to do when patients are increasing their exercise levels and/or travelling overseas, may be more relevant and fit-for-purpose for patients. PAAPs which focus narrowly on managing medicines and acute asthma, however, may be more suited to the more recently diagnosed. Critically, whether patients/parents are issued with PAAPs or *Living with Asthma* plans these must be patient-centred. Since asthma plans were first introduced, patients and parents have been actively encouraged to (self-) manage asthma [6, 7]. Asthma plan development/review therefore requires meaningful collaboration between professionals and patients/parents but our participants indicated that this was not routinely happening. Education is urgently required to encourage professional attitudinal and behavioural change. For instance, professionals need skills in negotiation and joint goal setting so PAAPs or *Living with Asthma plans* can be effectively developed with patients/parents. Professionals also require knowledge of patients/parents asthma management strategies and their information needs so this insight can inform development of asthma plans more aligned to patients/parents circumstances [13].

## Conclusions

Despite being recommended since the late 1980s, PAAPs are under-issued by professionals and under-used by patients internationally. Findings from this qualitative study provide yet more evidence of PAAP sub-optimal implementation in UK primary care. The multiple barriers to PAAP implementation reported by our participants that exist at individual (patient and professional) and organisational (practice) levels are symptomatic of a context that 25 years after PAAPs were first recommended is still not fully ready to consistently support their implementation. Multi-faceted interventions aimed at changing patient, professional and practice behaviours are required to facilitate development of a primary care context conducive to their implementation. Increasing the perceived value of PAAPs to professionals and patients is essential for their future use to increase. This could be facilitated by professionals issuing asthma plans that are more patient-centred and address the wider self-management issues of relevance to those living with this condition. This requires organisational change to create an environment that values communication skills that enable professionals to develop meaningful asthma plans in discussion with patients, and in which self-management is actively supported. The implementation of such multi-faceted approaches will warrant further investigation through future experimental studies.

## Additional file

Additional file 1: Table S1. Other examples of participant interview quotes. (PDF 63 kb)

## Abbreviations

(GP): General practitioner
(HN): Hospital nurse
(NHS): National health service
(N/A): Not applicable
(Pat): Patient
(PAAP): Personal asthma action plan
(PN): Practice nurse
(UK): United Kingdom
