# Supplementary material for: The ‘vicious cycle’ of personalised asthma action plan implementation in primary care: a qualitative study of patients and health professionals’ views
Source: BMC Fam Pract. 2015 Oct 21;16:145. doi: 10.1186/s12875-015-0352-4 (PMC4618358; doi:10.1186/s12875-015-0352-4)
Supplement: Additional file 1: Table S1. — Other examples of participant interview quotes. (PDF 63 kb) [file 12875_2015_352_MOESM1_ESM.pdf]

Additional table 1: Other examples of participant interview quotes

**Patients generally do not see the value of Personal Asthma Action Plans (PAAPs)**
'I don't think [a PAAP is] necessary at the moment …… when it boils down to it, what is an asthma plan actually going to do for people?' (Patient 1/group 1).
'If I was expected to take that along to the [asthma] appointment … If I knew that I had to take that along then I guess it would be something that I knew where it was and I was referring to it, it wasn't just at the back of the drawer' (Patient 2/group 1).
'There is an assumption [by health professionals] that I was given [the PAAP] years ago and I am still using it' (Patient 2/group 1).
Interviewer: 'have you ever been asked for your plan when go for a review? 'No. That is why I am saying it should probably have been updated. It's the old medication on it. (Patient 2/group 2).
Interviewer: 'so you hadn't heard of asthma action plans or self-management plans? Patient: No plans, just any plans, forget it! (Patient 1/group 3).
'It's really tatty [dog-eared] because I have had it some years now'. Interviewer: does the nurse ask to look at it when you get [your] review? 'No not always, but I proffer it' (Patient 2/group 3).
'[PAAPs] could help people but myself, I know what I am doing. [It would help] people who are not as knowledgeable about their condition …. or if they were going to a foreign country or because their [asthma] is more acute' (Patient 3/group 3).
'It's next to my bed, I think. I've got to be honest - I think I've stuck it in the drawer' (Patient 1/group 5).
'It [a PAAP] wouldn't prevent me getting asthma'. [It] could be useful for younger people and more severe cases….. It's good to monitor things but I think that would benefit other people than myself' (Patient 1/group 5).
**Professionals do not fully value PAAPs**
'I am not sure I've had anybody put down an asthma plan in front of me in recent times' (GP/group 1).
'I had a lady yesterday …. She came in with her plan and we did go over it. But, that's the first time ever that has happened' (PN2/group 2)
'You don't see them [patients] ever get their plan out' (GP1/group 2)
Interviewer: do you ask patients to being the plans to the review? 'I suppose we should but no, I don't truthfully…. But they wouldn't bring them, they won't even bring their

inhalers - I have to bring up a plan that I issued five years ago' (PN1/group 2).

'They [patients] can't find it - because they have had it for years' (PN2/group 2).

'If you asked me whether I think [PAAPs] are any good, I would have to say well, I don't know because [patients] have never come and told me about it. …… I don't see [PAAPs] as having an impact' (GP1/group 2).

Interviewer: do you think an asthma plan would be suitable for everybody? 'No'. (PN1/group 3).

Interviewer: 'do you ask to see PAAP at annual reviews?' 'They [patients] don't always [have it with them] and then I probably don't ask then' (PN1/group 3).

'If it's too complicated and too many words in it …. People won't or can't be bothered to read it… If it is all just bland writing they won't read it if it's just a piece of paper' (GP1/group 3).

'They [PAAPs] are not appropriate for everybody' (GP1/group 4).

'I don't give [a PAAP] to everybody because … ….. If there is not an interest there for them to take ownership of it there is not any point in doing [one]' (PN1/group 4).

'They patients] never bring it [PAAP]' (PN1/group 5).

'Some people do want it [a PAAP] and some people don't. Some are very interested and they really like it and others just don't seem interested. If they have had asthma for a long time and if they have just managed it without the use of a management plan before they don't really feel that they want one' (PN2/group 5).

'[The local NHS asthma plan] it's just like a document …it's big and like an A4 piece of paper that is going to disappear somewhere …., I know myself if I took that home it would be lost and it would be recycled' (PN1/group 5)

'People with asthma live very hectic normal lives and the vast majority of the time asthma is low down on their radar and you know you don't have your plan on their coffee table or on their kitchen table every single day of the year it's filed away somewhere and so they forget about it' (GP1/group 5).

'I don't think it [a PAAP] would do them [patients] any harm although …..the more pieces of paper and the more documents they have maybe the less likely they are to read them all' (GP1/other group).

'The chronic asthmatics …. I think it [a PAAP] probably would be put in the back of a drawer' (PN2/other group).

'[If patients are] only using their inhaler once every six months - we haven't given them an action plan' (PN3/other group).

Multiple multi-level barriers are reducing the value of PAAPs in primary care

Individual level barriers - patients (with some suggestions for how these can be overcome)

‘So many of these things are so wordy’ (Patient 1/group 1).

‘[It’s] important you are using your own words within it, so how you describe what happens to yourself. …. that’s the wording that you use and that should be in your plan because often the medical wording can be different and less sort of relevant. So, yeah, it’s literacy but the wording itself is important’ (Patient 2/group 1).

‘I wouldn’t like if it [the PAAP] just came through the post or something like that….. I would feel like every house …. had got one of these … to me it would be like junk mail’ (Patient 3/group 1).

‘Instead of the tick list on the computer [professionals should] say ‘this is personal to you, bring it along with you, we’ve got a copy here on the computer. Tell me – has there been any need to use it? ….You need to make it individual to the person …. and the next time you go back that happened to you and, if it didn’t work, you get it updated …. It’s not something set in stone’ (Patient 2/group 2).

‘People need to acknowledge their own asthma … before they could use things like plans because then they wouldn’t think the plan was relevant to them’ (Patient 1/group 4).

‘[PAAP development] should mainly come from the patient and when you meet with the nurse or doctor … there might be a section, a small section for them to fill in at the bottom…… Everybody is different … the doctor is there to oversee but the information is given by the patient, it has to be patient driven’ (Patient 1/group 5).

Interviewer: when you go for your asthma review does the PN ask to see your plan? ‘No, she hasn’t. I am 100% certain about that’ (Patient 1/group 5).

Individual level barriers - professionals (with some suggestions about how these can be overcome)

‘There is no real room to give guidance about steps …..I have got a lot of people who are on step one but who also have the ability to step up to two and step back down again….. It would be nice to say what action they should take … when they have stepped up … and stepped down, [for example] how long do they need to be symptom free before they step back down?’ (PN1/group 1).

‘It's a real shock when you find later on that an action that you thought someone was happy with and they were going to do, they have not done at all because of some block in there with a little bit more conversation you could have cleared that block…. It could be an information thing or a lifestyle thing …. And that block is there and you need to work with [the patient] to overcome that so they will then work with you and stick with the plan’ (PN 1/group 1).

‘I don’t know [whether every patient should have a PAAP] because I don’t know exactly whether they are effective or not…. I don’t know whether they are any good or not from my own experience’ (GP 1/group 2).

‘If they have had an exacerbation or they have been poorly controlled, I would go over it. You make a clinical judgement don’t you, whereas if someone is ticking along

year to year to year ….. I am not going to be re-doing [the PAAP]' (PN2/group 2).

'You are meant to multi-dose….but in the Asthma UK plan there is nothing really for that' (PN2/group 2).

'It was pointless for us to go over every single plan for every patient - there are so many patients' (GP1/group 2).

'I must admit if they have had one I don't re-issue it. I don't know if you are meant to, are you meant to? I don't know. No, because then you are just duplicating what they have had anyway' (PN2/group 2).

Interviewer: 'do you ask patients to bring back their plans for review?' 'No, not so much their plan but their peak flow diary' (Hospital Nurse/group 2).

'At [the annual review], hopefully we give a management plan – not always – but hopefully or usually, if they want one' (PN1/group 5).

'The short term one [local NHS asthma management plan], it gives you lots of words but not actually that much area for me to write in information particularly for that patient. The [local NHS asthma] symptom management plan, I try and make patients aware of not just the symptoms but their peak flow readings as well and …..but there is nowhere in this leaflet for the information about peak flows as well' (PN2/group 5).

'It takes me a long time to get through [some PAAPs]….. If [the PAAP] it's not easy to look through and to see what you need to find in it I think you know lots of information is just too much sometimes' (PN2/group 5).

'How many would I give an actual plan to? Maybe 1 in 10'. Very few, because …..they [patients] get this and it goes straight in the bin or it lies around' (PN1/other group).

'[Professionals] making [PAAPs] too medicalised' (PN3/other group).

Interviewer: 'do you give out asthma plans?' 'No I don't …no I don't have anything to do with action plans….. I have on occasions said have you increased your inhalers according to what's been suggested on the action plan, but I've never actually asked to see the action plan' (GP1/other group).

'I try and remember to ask them [patients] for it [their PAAP], it depends on where and when in the day it is and what else is going on. Sometimes it is just a matter of trying to get somebody sorted and then seeing them back' (GP2/other group).

'It is sometimes difficult to issue a written asthma management plan to somebody through an interpreter, and sometimes it is better to try and explain it to them and get them to explain it back to you' (GP2/other group).

'There is just too many [PAAPs]' (PN1/other group).

'The local NHS asthma management plans] I don't tend to use them, I did …. but you are printing these off, they are big bulky things, patients stuff them in their handbags

and I kind of thought … I don’t think they were fully used. So I don’t tend to use them now’ (PN2/other group).

Organisational barriers

‘The plan it's not in front of me and we've got no way of storing on their file that I'm aware of’ (GP 1/group 1).

‘They are time-consuming. Further down the line you may save time in that you will not have [patients] coming back but that is not [the case] at that particular point in time’ (PN1/group 4).

‘I never mention it to them and it’s probably because I don't know the patients who have a plan and those who don't. …….‘She [the PN] knows the patients who need [a PAAP] … and who they are appropriate for’ (GP1/group 4).

‘There is no point in doing [a PAAP] if you have don’t have the time to go through it properly and we [GPs] don’t have that kind of time’ (GP1/group 4).

‘It doesn’t come out on the letter asking them to bring it [to their annual asthma review appointment] so I tend to say to them do you have your one handy in the house from last year? (PN1/group 5).

‘It doesn’t actually say in the letter [to bring their PAAP to their review], I should get that re-worded because it asks them to bring their inhalers but not their management plan’ (PN2/group 5).

Interviewer: ‘do the GPs issue plans?’ ‘Probably not. I would think probably not – they will probably leave that to us’ (PN2/other group).

‘The GP partners would just tend to send the patient to me for action plans’ (PN3/other group).

‘That is one of the big problems - there are quite a few of them [PAAP], there are huge numbers you can download of the internet, there are local ones there are the Asthma UK ones there are so many of them and I am not sure - it’s quite confusing for physicians, it probably is a bit confusing for the patients because they do get different things from different people and if they move practices across they will get a [local NHS] action plan from one practice and then they move and suddenly they get an Asthma UK one or within the hospital they will get a different one potentially from there’ (GP2/other group).
